# Relation between Intensity of Biocide Practice and Residues of Anticoagulant Rodenticides in Red Foxes (*Vulpes vulpes*)

**DOI:** 10.1371/journal.pone.0139191

**Published:** 2015-09-29

**Authors:** Anke Geduhn, Jens Jacob, Detlef Schenke, Barbara Keller, Sven Kleinschmidt, Alexandra Esther

**Affiliations:** 1 Julius Kühn Institute, Federal Research Centre for Cultivated Plants, Institute for Plant Protection in Horticulture and Forests, Vertebrate Research, Münster, North Rhine-Westphalia, Germany; 2 University of Münster, Institute of Landscape Ecology, Münster, North Rhine-Westphalia, Germany; 3 Julius Kühn Institute, Federal Research Centre for Cultivated Plants, Institute for Ecological Chemistry, Plant Analysis and Stored Product Protection, Berlin, Germany; 4 Food and Veterinary Institute Braunschweig/Hannover, Lower Saxony State Office for Consumer Protection and Food Safety, Hannover, Lower Saxony, Germany; Ghent University, BELGIUM

## Abstract

Anticoagulant rodenticides (ARs) are commonly used to control rodent infestations for biocidal and plant protection purposes. This can lead to AR exposure of non-target small mammals and their predators, which is known from several regions of the world. However, drivers of exposure variation are usually not known. To identify environmental drivers of AR exposure in non-targets we analyzed 331 liver samples of red foxes (*Vulpes vulpes*) for residues of eight ARs and used local parameters (percentage of urban area and livestock density) to test for associations to residue occurrence. 59.8% of samples collected across Germany contained at least one rodenticide, in 20.2% of cases at levels at which biological effects are suspected. Second generation anticoagulants (mainly brodifacoum and bromadiolone) occurred more often than first generation anticoagulants. Local livestock density and the percentage of urban area were good indicators for AR residue occurrence. There was a positive association between pooled ARs and brodifacoum occurrence with livestock density as well as of pooled ARs, brodifacoum and difenacoum occurrence with the percentage of urban area on administrative district level. Pig holding drove associations of livestock density to AR residue occurrence in foxes. Therefore, risk mitigation strategies should focus on areas of high pig density and on highly urbanized areas to minimize non-target risk.

## Introduction

Commensal rodent populations are mainly regulated by anticoagulant rodenticides (ARs) [1] in plant protection as well as for the protection of hygiene, environmental health and to prevent damage to stored food and materials. ARs inhibit the blood clotting of all vertebrates [2,3], which causes a risk for non-target animals to ARs. Direct bait intake by non-target animals results in primary poisoning, which has been shown for several non-target rodent and shrew species [4–6], whereas secondary poisoning happens when predators ingest poisoned prey [7]. A reduction of red fox (*Vulpes vulpes*) population density has been shown in France after bromadiolone applications in open areas against water voles (*Arvicola terrestris*) [8,9]. In Canada, fox density was significantly lower in areas where ARs were used to control Richardson’s ground squirrels (*Spermophilus richardsonii*) than in control areas [10]. Such decreases of predator densities can be due to poisoning (primary or secondary) of predators or to poisoning of prey or a combination thereof.

Liver samples of carcasses are often used to screen for AR poisoning in wildlife because AR active substances accumulate in animal tissue—mainly in the liver [11], whereas plasma retention times are considerably shorter [12]. Worldwide, many studies investigated diurnal birds of prey and owls to assess secondary non target exposure to ARs (e.g. Scotland [13], Great Britain [14], USA [15], Canada [16], Spain [17], New Zealand [18], France [19]). Common buzzards (*Buteo buteo*) [20,21], red kites (*Milvus milvus*) [13,19,21] and barn owls (*Tyto alba*) [22,23] are regularly exposed to ARs and mainly prey on small mammals [24,25]. Wildlife poisoning or exposure to ARs has also been shown in terrestrial predators like polecats (*Mustela putorius*) [26], stoats (*Mustela erminea*) and weasels (*Mustela nivalis*) [27,28].

AR residues had been quantified in feces of red foxes [29] and liver samples (e.g. France [20], USA [30,31], Spain [17]). Field application of ARs can be a source of secondary poisoning in predators [20]. An association of AR exposure to urban area has been shown in bobcats (*Lynx rufus*) [32] and some predatory bird species in Spain [33]. Nevertheless, most studies monitored ARs in predator carcasses without consideration of environmental drivers of exposure.

Regulation of rodenticide usage varies among countries. In Germany, only difenacoum was authorized in plant protection products during the period in which foxes were collected for the present study, but only for use in and around buildings to protect stored products, whereas eight ARs (see below) were authorized for biocidal use. Therefore, exposure of predators to ARs via field application for plant protection should not have occurred. Three registered substances are first generation ARs (FGARs: chlorophacinone, coumatetralyl and warfarine) and five are second generation ARs (SGARs: brodifacoum, bromadiolone, difenacoum, difethialone and flocoumafen). SGARs have a higher toxicity to vertebrates and persist longer in tissues than FGARs [18,34]. This could lead to an enhanced risk of secondary poisoning for predators from SGAR, which has been demonstrated for several species [18,35]. ARs are used as biocides in and around buildings at farms when rodents occur, to prevent contact to livestock, animal food and stored products. In urban areas Norway rats (*Rattus norvegicus*) are often controlled in sewage systems. Furthermore, Norway rat and house mice (*M*. *musculus*) infestations can be found for instance in all sectors of the food industry (including restaurants and supermarkets), living quarters and public parks.

Red foxes are carnivores with a wide distribution [36]. They inhabit urban environments [37,38] as well as farmlands [37,39]. In farmland areas red foxes mainly prey on small mammals and birds [39,40]. When animal food is stored on the ground it is easily accessible to rodents [41], suggesting more intense AR usage on farms with many open food resources. The diet of urban foxes is dominated by scavenged meat, but commensal rodents are hunted as well [42]. Therefore, red foxes could be at risk to ingest poisoned prey during rodent baiting with ARs in urban and rural situations and seem a suitable study species for analyzing the effect of environmental drivers (i.e. urban area and livestock) of exposure of predators to ARs.

The aim of our study was to identify factors that influence the exposure of foxes to ARs. We analyzed residue occurrence of ARs in fox liver samples in relation to the intensity of livestock holding and the percentage of urban area. We hypothesized that increased livestock density and increased urban area result in higher AR usage, due to increased rodent manifestations and common AR application resulting in high exposure of foxes to ARs. Furthermore we expected livestock species that are kept in indoor feedlots on a farm to be more associated with increased AR application than species kept outdoors. Information about environmental factors that are associated with FGAR and SGAR exposure in predators can aid optimizing risk assessment and developing risk mitigation strategies.

## Materials and Methods

### Sample sources

**Fig 1:**
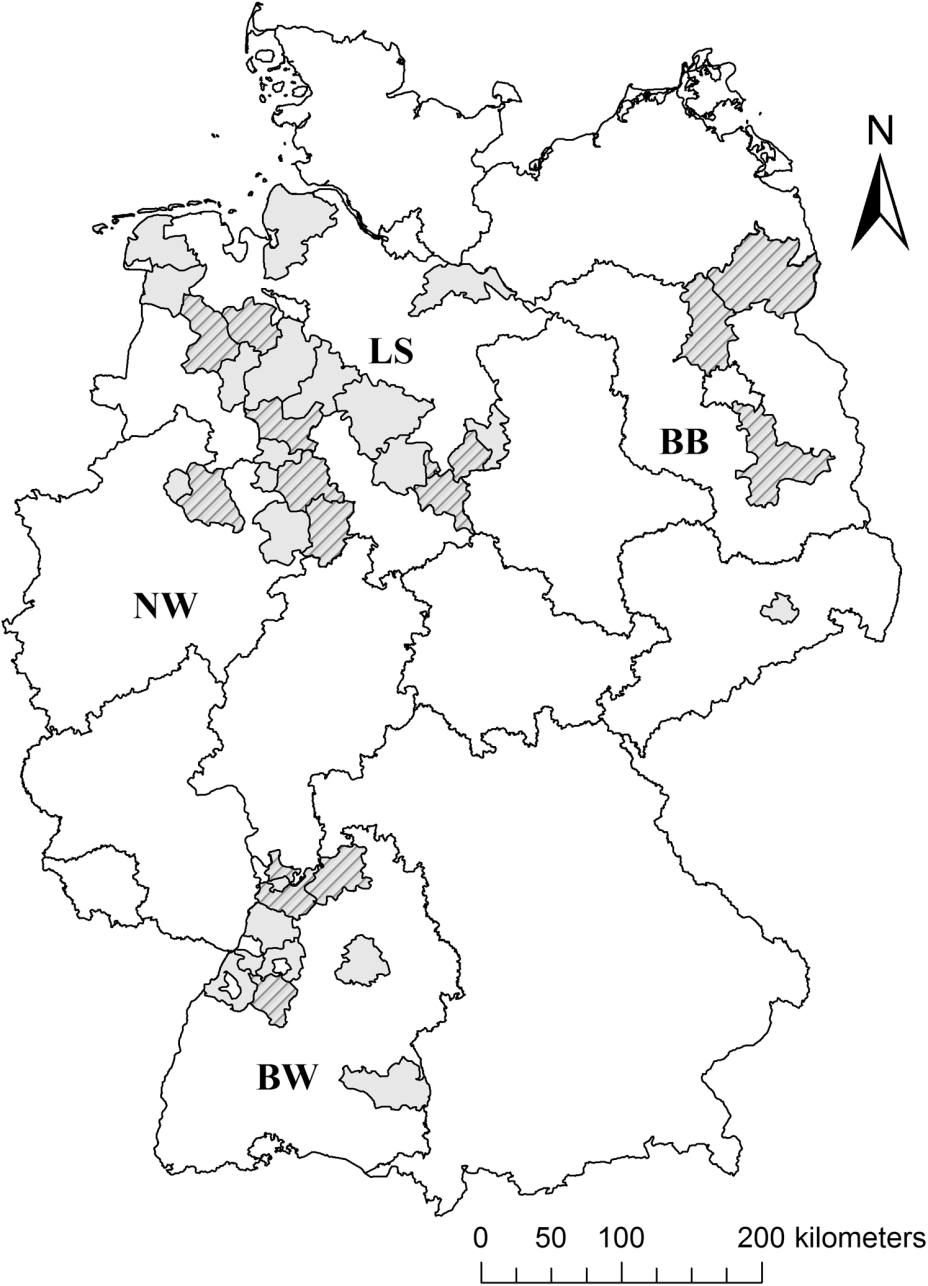
Sources of red fox (*Vulpes vulpes*) liver samples used for local parameter association. Samples originated from 35 German administrative districts (shaded areas) within the federal states LS (Lower Saxony), NW (North Rhine-Westphalia), BB (Brandenburg) and BW (Baden-Wuerttemberg). Districts that provided at least 5 liver samples (striped areas) were used for local parameter analysis. The graphic is based on http://www.bkg.bund.de/nn_167688/SharedDocs/Download/DE-Karten/Verwaltungskarte-Deutschland-BRK-DIN-A3,templateId=raw,property=publicationFile.pdf/Verwaltungskarte-Deutschland-BRK-DIN-A3.pdf (accessed 15 January 2015) adjusted using ArcGIS 10.0.

Liver samples from 331 red foxes derived from 35 administrative districts were mainly provided by veterinary institutes of the German federal states Brandenburg, Baden-Wuerttemberg, Lower Saxony and North Rhine-Westphalia in 2012 and 2013. 301 foxes were individuals found dead or shot in the states’ rabies monitoring scheme. An additional 30 foxes originated from the Warendorf district (North Rhine-Westphalia) were provided by a taxidermist. These foxes were dissected at Julius Kühn Institute. No information was available on age or sex of foxes. For pathogen disinfection (e.g. *Echinococcus multilocularis*) liver samples were frozen for at least one week at -80°C and stored afterwards at -20°C. Samples (N = 303) from districts (N = 14) that provided at least 5 liver samples (Fig 1) were used to determine associations between AR residue occurrence and local parameters of the districts.

### Anticoagulant rodenticide residue analysis

The analysis of liver samples is described in detail by Geduhn et al. [5]. Shortly summarized, subsamples of about 1.5 g liver were taken from defrosted liver samples and spiked with a surrogate mixture and homogenized into an ice bath with methanol/water. Interfering substances were removed by solid supported liquid extraction on a diatomaceous earth column. The quantification of the eight ARs (brodifacoum, bromadiolone, chlorophacinone, coumatetralyl, difenacoum, difethialone, flocoumafen and warfarin) were realised by LC-MS/MS in electrospray ionization negative mode. The calibration standards including internal standards (chlorophacinone-d_4_ and warfarin-d_5_) and surrogates (acenocoumarol, phenprocoumon, diphacinone-d_4_ and coumachlor; 0.1 to 100 ng/ml; r^2^ > 0.99) were solved in methanol:water (1:1). The limits of detection with a signal to noise ratio of > 3:1 were between 0.001 μg/g for coumatetralyl, 0.002 μg/g for warfarin, difenacoum, 0.003 μg/g for brodifacoum, bromadiolone and 0.005 μg/g for difethialone, flocoumafen and chlorophacinone. Spectra comparison between sample and references based on Enhanced Product Ion-spectra was done additionally. Recovery rates based on spiking clean turkey (*Meleagris gallopavo*) liver (0.2 μg/g, N = 4) ± standard deviation were for chlorophacinone 83% ±14%, warfarin 118% ±4%, coumatetralyl 100% ±6%, difenacoum 78% ±7%, bromadiolone 77% ±4%, brodifacoum 58% ±6%, flocoumafen 65% ±4% and difethialone 41% ±7% and for the surrogate acenocoumarol 112% ±5%, diphacinone-d_4_ 106% ±9%, phenprocoumon 101% ±1% and coumachlor 91% ±2%. Concentrations are μg AR active substance per g liver wet weight throughout and were extrapolated to 100% based on recovery rates in clean liver samples.

### Data preparation and statistical analysis

To compare the occurrence of FGARs and SGARs in fox liver samples, the percentage of residue occurrence and the median concentration in individuals that carried a residue for each active substance was calculated (Table 1). Two sided Welch t-tests were used to test for differences in occurrence and concentrations of ARs between FGAR and SGAR because values were normally distributed but variances and sample sizes were unequal. Residue concentrations were classed in five groups (0, >0<0.2, ≥0.2<0.8, ≥0.8<2.0, ≥2.0 μg/g) according to biological effects from concentrations that are known from the literature [20,29] and are discussed.

**Table 1:** Residues of anticoagulant rodenticides (ARs) in red fox liver samples. Number (N) of red foxes (*Vulpes vulpes*) containing residues and concentrations in μg/g of residues from AR positive individuals are stated for all eight analyzed ARs. % refers to the total number of 331 samples.

			Residues in positive individuals [μg/g]			
	N	%	Mean	Median	Min.	Max.
*FGARs*						
Chlorophacinone	1	0.3	0.013			
Coumatetralyl	19	5.7	0.130	0.025	0.001	0.891
Warfarin	2	0.6	0.010	0.010	0.008	0.012
*SGARs*						
Brodifacoum	151	45.6	0.267	0.091	0.010	2.433
Bromadiolone	125	37.8	0.185	0.061	0.004	1.574
Difenacoum	37	11.2	0.087	0.029	0.010	0.774
Difethialone	26	7.9	0.099	0.065	0.017	0.327
Flocoumafen	46	13.9	0.102	0.048	0.008	0.838

Livestock parameters and the percentage of urban area were obtained from GENESIS-online-database [43]. Livestock units (1 livestock unit = 500 kg body weight) from a survey in 2010 [43] were used to calculate livestock density as livestock units per area (100 ha) for each relevant district. Data were pooled for all livestock larger than chickens in one model. Furthermore, the number of livestock individuals per 100 ha for cattle, pigs and sheep (from a survey in 2010) and the number of laying hens per 100 ha (from a survey in 2007, because no more recent data were available) were analyzed for associations to residue occurrence of pooled ARs and brodifacoum.

The first aim was to investigate if livestock density and the percentage of urban area within German districts affect the occurrence of residues of pooled ARs and the four most prevalent AR substances found in red foxes (brodifacoum, bromadiolone, difenacoum and flocoumafen). We used binomial linear mixed models to screen data for associations between AR occurrence with livestock density and the percentage of urban area of 14 German districts (shaded in Fig 1) with one model per AR substance and one where we pooled all ARs. There were at least 5 liver samples from red foxes per district. The depending variable was a combined vector of the number of foxes that contained and did not contain residues (cbind function) per German district (N = 14). We added the federal state as random factor in both models (presence/absence of AR ~ livestock density + urban area + (1|federal state)). For modeling we used the lme4 package [44]. The variance inflation factor (VIF) was calculated using the vif.mer function for the fixed factors of each model, but no multi-collinearity occurred between livestock density and urban area in any model (VIF<3, referring to [45]).

As livestock density was associated to the residue occurrence of pooled ARs and of brodifacoum, the second aim was to test if specific livestock species drive these associations. Therefore, we designed two models containing cattle, pig, sheep and laying hen density as explanatory variables and pooled ARs and brodifacoum residue occurrence (combined counts of AR positive and negative individuals per district) as depending variable. The variable with the highest VIF (at least >3) was excluded from the model. VIFs of factors from adapted models were all <3. The final model was selected stepwise by AIC (Akaike information criterion) comparison and resulted in: 1) presence/absence of pooled ARs ~ pig density + (1|federal state) and 2) presence/absence of brodifacoum ~ pig density + (1|federal state).

All covariates were standardized before modeling by subtracting the mean and dividing by the standard deviation to reduce associations between parameter estimates. R² values are not provided by lme4. We calculated pseudo r-square values from linear regression of observed vs. predicted values to derive power of the mixed models. All statistical analyses were conducted with R 3.1.3 [46]. Level for significance was p≤0.05.

## Results

### AR residues in fox liver on animal level

198 of 331 liver samples (59.8%) from red foxes contained residues of at least one AR. 128 samples (38.7%) contained more than one active substance, 70 samples (21.1%) contained two active substances, 44 three (13.3%), 6 four (1.8%), 7 five (2.1%) and one sample (0.3%) contained six different AR active substances. Chlorophacinone was detected only once (0.3%) while 151 individuals (45.6%) contained residues of brodifacoum (Table 1). Median values of residue concentrations of AR positive samples varied between 0.010 μg/g for warfarin and 0.091 μg/g of brodifacoum (Table 1).

**Fig 2:**
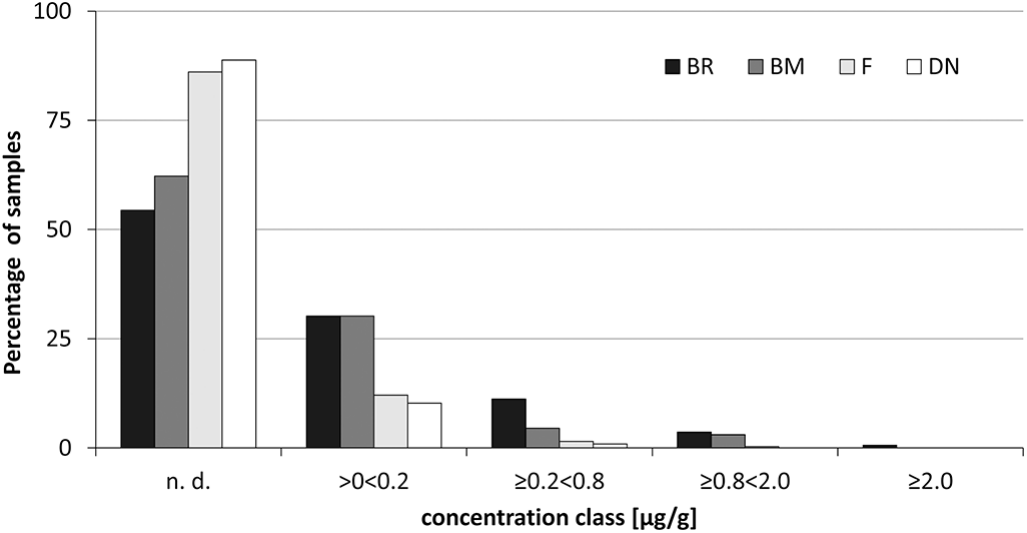
Residues of anticoagulant rodenticides in red fox liver samples. Percentage of samples containing no residues (n. d.), brodifacoum (BR), bromadiolone (BM), flocoumafen (F), difenacoum (DN) in four concentration classes.

Brodifacoum and bromadiolone residues were found most often and residue concentrations were highest for these active substances (Table 1 and Fig 2). Two individuals contained brodifacoum residue concentrations >2.0 μg/g, whereas all active substances occurred as least once with a concentration >0.2 μg/g except chlorophacinone and warfarin. Residue concentrations >0.2μg/g occurred in 51 samples (15.4%) containing brodifacoum and in 25 samples (7.6%) containing bromadiolone. Residues >0.8 μg/g (including concentrations >2.0 μg/g) almost exclusively occurred in samples with brodifacoum (N = 12; 3.6%) or bromadiolone (N = 10; 3.0%) residues (Fig 2). Residues of flocoumafen and difenacoum rarely occurred in all concentration classes and only sporadically at concentrations >0.2 μg/g (Fig 2).

**Fig 3:**
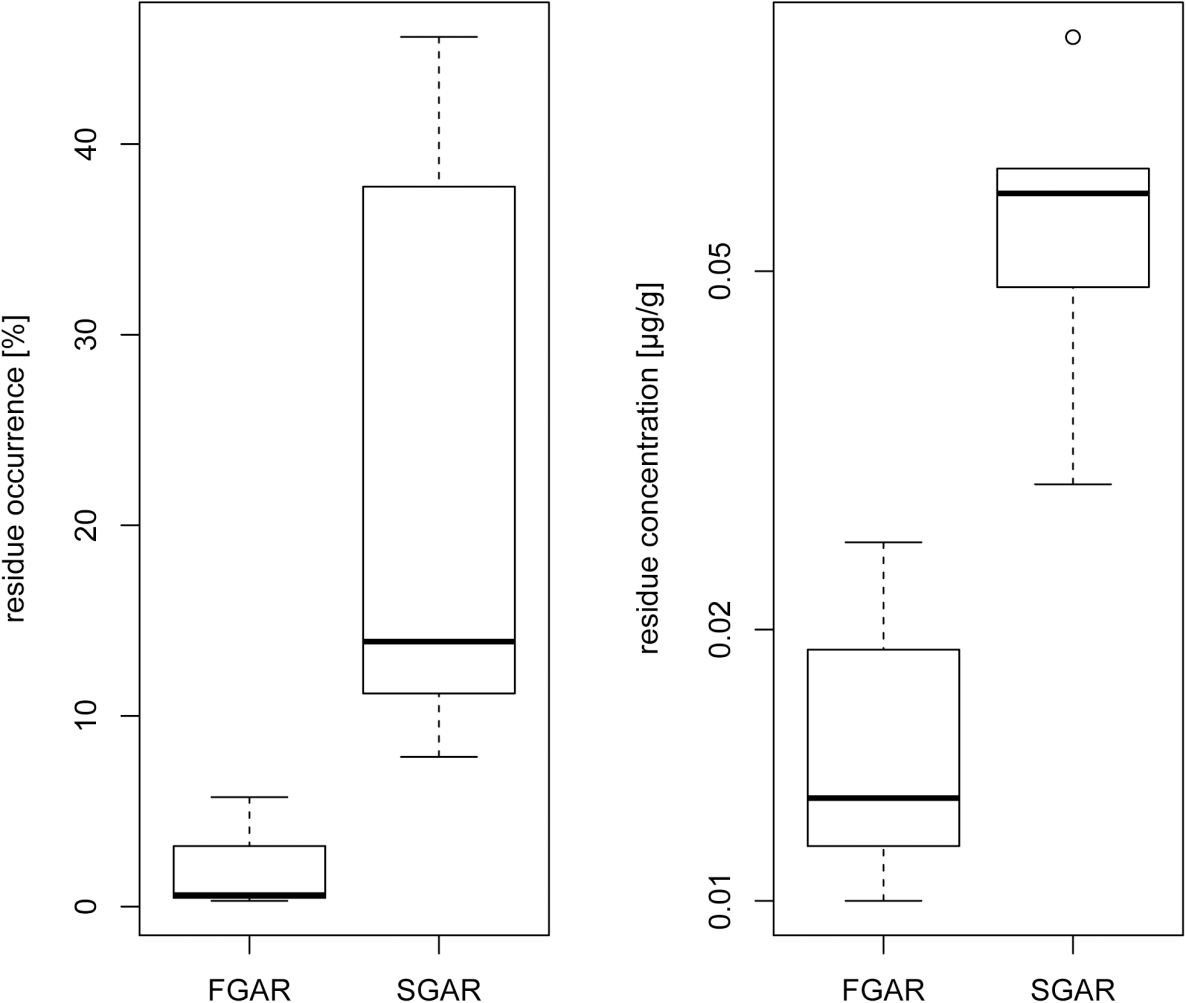
Residues of first (FGARs) and second (SGARs) generation anticoagulant rodenticides in fox liver samples. FGARs include chlorophacinone, coumatetralyl and warfarin; SGARs include brodifacoum, bromadiolome, difenacoum, difethialone and flocoumafen. Left: FGAR (N = 3) and SGAR (N = 5) occurrence (one percentage value per active substance); Right: FGAR (N = 3) and SGAR (N = 5) residue concentrations (median residue concentration per active substance).

SGARs (brodifacoum, bromadiolone, difenacoum, difethialone, flocoumafen) residues occurred more often than FGARs (chlorophacinone, coumatetralyl and warfarin) (Table 1, Fig 3 (left); N FGARs = 3, N SGARs = 5, t = -2.67, p = 0.05). Median concentrations were lower in FGARs than in SGARs (Fig 3 (right): median: N FGARs = 3, N SGARs = 5, t = -3.82, p = 0.011).

### Spatial distribution of AR residues

The occurrence of AR residues was determined in 14 German districts (Table 2, Fig 1). Residue occurrence of brodifacoum varied from 13 to 100% and was the lowest in districts of the federal state Baden-Wuerttemberg (mean: 34%) and the highest in North Rhine-Westphalia (mean: 79%) compared to all other federal states. Occurrence of bromadiolone residues varied between 0 and 80% and was the lowest again in districts of Baden-Wuerttemberg (mean: 16%) and the highest in North Rhine-Westphalia (mean: 52%). Median concentration of brodifacoum was remarkably high in the district of Minden-Luebbecke and low in the Rhein-Neckar district, whereas the concentration of bromadiolone was the highest in Rhein-Neckar (Table 2) compared to all other districts.

**Table 2:** Residue distribution of anticoagulant rodenticides (ARs) in German districts. Number (N) of red foxes (*Vulpes vulpes*) containing anticoagulant rodenticide (AR) residues in their livers and concentrations in μg/g of individuals where ARs were present: all anticoagulants (AR), brodifacoum (BR) and bromadiolone (BM). German districts are located in four federal states (Brandenburg: BB, Baden-Wuerttemberg: BW, Lower Saxony: LS, North Rhine-Westphalia: NW).

			Ind. containing residues				Median concentration [μg/g]		
State	District	N	AR	%	BR	BM	AR	BR	BM
BB	Dahme-Spreewald	39	17	43.6	11	12	0.062	0.052	0.018
	Oberhavel	41	22	53.7	16	10	0.152	0.073	0.041
	Uckermark	39	23	59.0	16	19	0.190	0.125	0.055
BW	Calw	12	2	16.7	2	0	0.057	0.057	-
	Neckar-Odenwald	29	10	34.5	10	3	0.085	0.047	0.045
	Rhein-Neckar	28	20	71.4	14	11	0.148	0.029	0.192
LS	Cloppenburg	5	5	100.0	5	2	0.212	0.088	0.052
	Goslar	26	14	53.8	9	11	0.194	0.117	0.144
	Oldenburg	8	7	87.5	7	6	0.242	0.147	0.104
	Wolfenbuettel	15	6	40.0	2	3	0.068	0.055	0.014
NW	Hoexter	6	5	83.3	5	1	0.287	0.060	0.057
	Lippe	21	18	85.7	16	11	0.367	0.167	0.088
	Minden-Luebbecke	5	5	100.0	5	4	0.864	0.412	0.062
	Warendorf	29	23	79.3	16	17	0.300	0.512	0.035

### Effects of local parameters on AR residue occurrence

**Fig 4:**
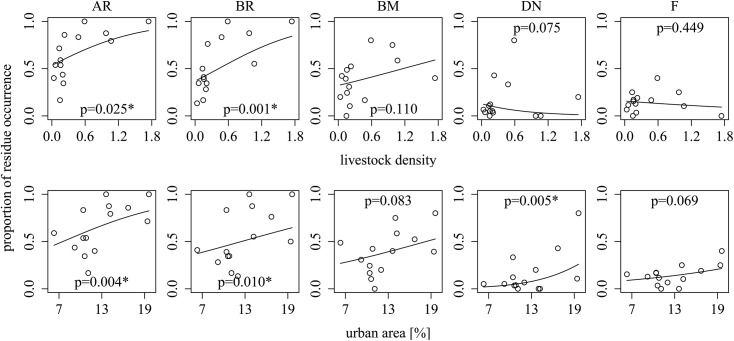
Associations between local parameters and residue occurrence of anticoagulant rodenticides (ARs). Relations between residue occurrence (y-axis [%]) in red fox (*Vulpes vulpes*) livers and livestock density as well as the percentage of urban area per district in Germany (N = 14) are displayed for pooled anticoagulants (AR; any of all eight analyzed ARs), brodifacoum (BR), bromadiolone (BM), difenacoum (DN) and flocoumafen (F). Lines represent model predictions. Significant associations are marked with *.

Livestock density was positively associated with the occurrence of pooled ARs and the occurrence of brodifacoum in the 14 districts (Table 3, Fig 4). AR occurrence ranged from 79 to 100% in samples from districts with a livestock density above 0.45, whereas below 0.45 AR occurrence varied between 17 and 86% but mostly between 40 and 65% (Fig 4). Brodifacoum occurrence in foxes from districts with livestock densities above 0.45 varied from 55 to 100% whereas below that threshold brodifacoum occurred in 13 to 76% of samples per district (Fig 4).

**Table 3:** Model results of associations between residue occurrence and local parameters. Residue occurrence (No. of pos and neg. individuals) of any anticoagulant rodenticide (AR) and for each active substance (brodifacoum–BR, bromadiolone–BM, difenacoum–DN and flocoumafen–F) in 14 districts was screened for associations with local parameters. Model results and pseudo r² values, including fixed and random factors, are given. Significant associations are marked with *.

Substance	R²	Parameter	Estimate	SE	P
*Livestock density / urban area*					
AR	0.814	Intercept	0.667	0.233	0.004*
		Livestock density	0.594	0.264	0.025*
		Urban area	0.488	0.171	0.004*
BR	0.641	Intercept	0.022	0.135	0.874
		Livestock density	0.630	0.193	0.001*
		Urban area	0.322	0.125	0.010*
BM	0.548	Intercept	-0.463	0.248	0.062
		Livestock density	0.317	0.198	0.110
		Urban area	0.317	0.183	0.083
DN	0.735	Intercept	-2.532	0.507	<0.001*
		Livestock density	-0.693	0.388	0.075
		Urban area	0.822	0.292	0.005*
F	0.385	Intercept	-1.832	0.183	<0.001*
		Livestock density	-0.170	0.252	0.499
		Urban area	0.295	0.162	0.069
*Livestock species*					
AR	0.600	Intercept	0.624	0.153	<0.001*
		Pig density	0.827	0.218	<0.001*
BR	0.482	Intercept	-0.065	0.129	0.616
		Pig density	0.610	0.163	<0.001*

The percentage of urban area was positively associated with pooled ARs, brodifacoum and difenacoum occurrence (Table 3, Fig 4). In districts with urban areas <13%, AR residues occurred in 17 to 83% of samples, whereas AR residue occurrence varied from 71 to 100% in samples from districts with urban area >13% (Fig 4).

In a second step, density (No. individuals per area) of cattle, pig, sheep and laying hens were tested for associations with pooled AR residues and brodifacoum. Cattle density was highly collinear to other livestock density (verified by the variance inflation factors VIF >3) in both models and was therefore excluded from the analysis. Pig density was positively associated to residue occurrence of pooled ARs and brodifacoum residues (Table 3). Sheep and laying hen densities were excluded by model simplification through AIC.

## Discussion

Our large-scale study provides clear evidence for AR exposure in red foxes across Germany. Residues of brodifacoum and bromadiolone were most common and at the highest concentration. ARs occurred in foxes from all German districts; therefore, wildlife contamination with ARs is not a local problem. All ARs authorized in the EU/Germany for use in either biocidal products or plant protection products could be found in at least one liver sample. Residues of SGARs were considerably more common than those of FGARs and were detected at higher residue concentrations.

Accumulation could explain those differences because SGARs have a remarkably longer persistence in animal tissues than FGARs [12]. Another possible explanation is a more common usage of SGARs than FGARs, which is indicated by the fact, that more SGAR than FGAR products (425 versus 82) are authorized in Germany [47]. Resistance to FGARs [48–50] has led to the development and use of SGARs. For example SGARs are known to be applied much more frequently than FGARs in Scotland [13]. In a farmer survey in the Münsterland (Germany), brodifacoum was the active substance used most often for biocidal rodent management [5], most likely due to the fact that resistances against FGARS as well as some SGARS have been reported for this region. Additionally, brodifacoum is a rodenticide active substance with a long persistence in liver tissue [34] resulting in a longer time window when residues can be detected. This could explain the highest occurrence and concentration of brodifacoum residues in foxes in the present study. The rare occurrence of FGARs in fox liver samples (5.4% of all residues) suggests that these active substances do not play an important role in secondary poisoning with ARs in red foxes in Germany. In contrast, the occurrence of SGARs (94.6% of all residues) indicates high exposure of predators and increased risk of poisoning due to high toxicity and persistence.

AR usage is common at livestock farms, because often livestock food is easily accessible to commensal rodents and there are plentiful places to hide and rest [51], resulting in high population densities of rodents. Red foxes in farmland mainly prey on rodents [39] and are therefore at risk of secondary poisoning in these areas, which could explain the positive association between AR and brodifacoum residue occurrence and livestock density we found. The results show a clear association of livestock density and AR residues in foxes suggesting pronounced AR usage associated with livestock production. The latter seems plausible but cannot be confirmed because no detailed data are available about AR application in animal husbandry. There are almost no publications that investigate the effect of local parameters such as landscape and land use on residues of ARs in red foxes, except Tosh et al. [52], who demonstrated higher exposure of red foxes to ARs in lowlands than in “less favourable areas” suggesting more intense AR application in agriculturally used lowlands.

Pig density was positively associated with AR and brodifacoum residue occurrence whereas sheep density and laying hen density were not associated to pooled AR or brodifacoum occurrence. In Germany management of commensal rodents is mandatory in pig husbandry [53] but not in cattle, laying hen or sheep holding, suggesting more intense AR usage on pig farms. This is in line with the positive associations of pig density and residue occurrence. Nevertheless, rats regularly occur on cattle farms as well, because of easy access to food and nest sites. Density of cattle was collinear to other livestock species and was removed from both models (pooled AR and brodifacoum) before model selection by AIC. Therefore, cattle density could also explain the association of pig density and residue occurrence of pooled ARs and brodifacoum. In contrast to cattle and pigs that are often kept indoors in feedlots sheep are commonly kept free-range on pastures. AR application in sheep husbandry therefore could be less common, because the application of biocidal ARs is often restricted to the use in and around buildings and less food is available for commensal rodents on pastures. This is reflected in the lack of an association between sheep livestock density and AR occurrence. Chicken holding was also expected to represent a source of ARs, but no association was found for laying hens. The lack of an association may be due to the restriction of the available data to laying hens, which represent only a subset of chicken holding.

Beside agricultural usage of ARs they are regularly applied in urban areas to control commensal rodents. Commensal rodents occur in a wide range of habitats in urban areas such as sewage systems, waste dumps, in parks and gardens, within the food industry (supermarkets, bakeries, restaurants etc.) and in apartment buildings and houses, where waste and food is accessible to rodents [54,55]. Our results suggest a risk for foxes to ingest ARs in these areas. Pooled ARs, brodifacoum and difenacoum residue occurrence in German districts associated positively with the percentage of urban area. The diet of red foxes in urban and periurban areas of Zurich consists of 11% rodents and 10% of them are commensal rodents [42], which could explain the uptake of ARs by urban foxes. In Eastern Germany urban foxes mainly prey on waste, but commensal rodents are taken also and in higher frequencies than in rural areas, where *Microtus* species are the predominant rodent taxa [37]. Brodifacoum and difenacoum were the main ARs detected in foxes in urban areas, which is similar to results observed in bobcats in California [32] and predatory birds in Spain [33].

The high variation of AR occurrence among German districts suggests that also other factors than livestock density and urbanization influence AR distribution in predators. Further research is needed for a better understanding of the pathways of AR exposure in predators.

In other studies residues of ARs were found in red foxes, but active substances and concentrations differ greatly. In the UK, France and Spain bromadiolone residues are most commonly found in foxes but liver concentrations vary extremely [17,20,52]. Bromadiolone concentrations we found (median: 0.061 μg/g and a range from 0.0004 to 1.574 μg/g) were remarkably lower than those in France (0.800 to 6.900 μg/g [20]), but similar to those found in the UK (0.004 to 1.781 μg/g [52]) and Spain (mean 0.155 μg/g [17]). High concentrations found in France could result from extensive field usage there [20], which is prohibited in Germany. Brodifacoum residues occur less often than bromadiolone residues in Spain and the UK [17,52], whereas brodifacoum residues occurred in all of 5 red fox samples in the US [22,56]. Brodifacoum concentrations in the present study were higher than those found in the UK (0.003 to 0.654 μg/g [52]) suggesting higher risk for predators in the German environment. However, the comparative assessment of results among studies concerning secondary poisoning of non-target predators is difficult due to different usage patterns of AR, local situations and AR residue analysis methods. Furthermore, residues in our study were corrected for recovery rates, making concentrations comparable between active substances but this was not done in all previous studies (e.g. [17], [52]). If residue concentrations of brodifacoum are not corrected for recovery rates, our findings are similar to those reported by Tosh et al. [52].

Nevertheless, residue concentrations may provide rough information on biological effects in non-target animals. Sage et al. [29] found liver residues of about 2.0 μg/g in red foxes 24 to 26 days after feeding them with bromadiolone poisoned rodents. Two out of four foxes were suspected to have died without an injection of the antidote vitamin k. Concentrations of bromadiolone in livers of red fox carcasses in France ranged from 0.8 to 6.9 μg/g in individuals with clinically confirmed poisoning signs, but a threshold of 0.2 μg/g was suspected for biological effects [20]. We found 7.6% red foxes containing bromadiolone concentrations ≥0.2 μg/g and 3.0% carried bromadiolone residues ≥0.8 μg/g suspecting toxicological effects for these individuals. No fox had bromadiolone residues above 2.0 μg/g. To our knowledge there are no studies concerning biologic effects of brodifacoum residues in fox livers. Brodifacoum is more toxic to mammals than bromadiolone [30,57]. Therefore, using the thresholds for bromadiolone for brodifacoum should underestimate effects. 15.4% of tested red foxes had brodifacoum residues ≥0.2 μg/g, 3.6% at concentrations ≥0.8 μg/g and 0.6% at >2.0 μg/g. Therefore, biological effects of ARs were most likely through brodifacoum, although we could not screen fox carcasses for haemorrhaging to confirm this suggestion. 27.5% of foxes had pooled SGAR residue concentrations above 0.2 μg/g, including 35 samples (10.6%) with residues higher than 0.8 μg/g and 5 (1.5%) samples with even more than 2.0 μg/g. Accumulation of different AR active substances could enhance biological effects, but effects of interactions between substances are unknown.

In total 20.2% of samples contained a particular SGAR with a concentration higher than 0.2 μg/g. Residues of flocoumafen and difenacoum occurred considerably less often than brodifacoum and bromadiolone and concentrations above 0.2 μg/g occurred in only 6 and 4 samples respectively, a concentration >0.8 μg/g was measured once for flocoumafen. LD_50_ values of flocoumafen and difenacoum for house mice are between the values of brodifacoum and bromadiolone [12]. Therefore, biological effects on red foxes seem less common but possible. Residue concentrations >0.2 μg/g were even rarer for all other active substances and a concentration >0.8 μg/g of AR occurred in only one sample (coumatetralyl), which demonstrates that the biological relevance for non-target predators especially of brodifacoum and bromadiolone is higher than the relevance of flocoumafen and difenacoum. Concentrations of other AR substance were even lower. Based on such low residue concentration and occurrence it is suggested that there was low risk imposed on non-target predators by these ARs in the system considered here.

## Conclusion

Residues of ARs such as brodifacoum and bromadiolone in red foxes are widespread in Germany, which reflects the widespread use of these active substances as biocidal rodenticides in Germany. Our study highlighted that foxes carry residues of SGARs more frequently and in higher concentrations than FGARs. Therefore, risk mitigation strategies should consider especially SGARs when these compounds are used in the biocide sector. The occurrence of AR residues associated positively with livestock density in general and with that of pigs in particular, indicating that ARs do not affect non-target terrestrial predators similarly in all land use types. This is especially the case for the highly persistent SGAR brodifacoum that is commonly used in animal holding. Our results are consistent to the hypothesis that indoor livestock feedlots are a source for AR exposure to non-target predators, in our case mainly from pig (or cattle) production. As a conclusion, risk mitigation strategies are needed for AR application in farmland. In contrast to brodifacoum residues that were associated to livestock density as well as to the percentage of urban area difenacoum residues seemed mainly to originate from urban areas. 7.6% of red foxes in our study may have been biologically affected by bromadiolone, based on the results of Sage et al. [29] and Berny et al. [20] and possibly even more by brodifacoum. More detailed information about the relation of AR uptake and liver concentrations are required to make precise assessments also for other AR substances. Research on risk mitigation strategies should focus on application methods in areas with high livestock density as well as on urban areas.

## Supporting Information

S1 Table Residues of anticoagulant rodenticides [μg/g] (recovery corrected) of 331 analyzed red fox (*Vulpes vulpes*) liver samples from administrative districts in Germany. (PDF)
