# Supplementary material for: Relation between Intensity of Biocide Practice and Residues of Anticoagulant Rodenticides in Red Foxes (Vulpes vulpes)
Source: PLoS One. 2015 Sep 29;10(9):e0139191. doi: 10.1371/journal.pone.0139191 (PMC4587841; doi:10.1371/journal.pone.0139191)
Supplement: S1 Table — (PDF) [file pone.0139191.s001.pdf]

S1 Table. Residues of anticoagulant rodenticides [µg/g] (recovery corrected) of 331 analyzed red fox (*Vulpes vulpes*) liver samples from administrative districts in Germany

Fox sample	Administrative District	Brodifacoum	Bromadiolone	Chlorphacinone	Coumatetralyl	Difenacoum	Difethialone	Flocoumafen	Warfarin
1	Aurich	0.029	0.123	0.000	0.000	0.000	0.000	0.000	0.000
2	Biberach	0.028	0.247	0.000	0.000	0.000	0.000	0.000	0.000
3	Biberach	0.060	1.068	0.000	0.000	0.000	0.000	0.000	0.000
4	Bielefeld	0.019	0.000	0.000	0.000	0.044	0.000	0.000	0.000
5	Calw	0.000	0.000	0.000	0.000	0.000	0.000	0.000	0.000
6	Calw	0.000	0.000	0.000	0.000	0.000	0.000	0.000	0.000
7	Calw	0.000	0.000	0.000	0.000	0.000	0.000	0.000	0.000
8	Calw	0.000	0.000	0.000	0.000	0.000	0.000	0.000	0.000
9	Calw	0.000	0.000	0.000	0.000	0.000	0.000	0.000	0.000
10	Calw	0.000	0.000	0.000	0.000	0.000	0.000	0.000	0.000
11	Calw	0.000	0.000	0.000	0.000	0.000	0.000	0.000	0.000
12	Calw	0.000	0.000	0.000	0.000	0.000	0.000	0.000	0.000
13	Calw	0.000	0.000	0.000	0.000	0.000	0.000	0.000	0.000
14	Calw	0.000	0.000	0.000	0.000	0.000	0.000	0.000	0.000
15	Calw	0.017	0.000	0.000	0.000	0.000	0.000	0.000	0.000
16	Calw	0.097	0.000	0.000	0.000	0.000	0.000	0.000	0.000
17	Cloppenburg	0.071	0.000	0.000	0.000	0.000	0.000	0.000	0.000
18	Cloppenburg	0.088	0.000	0.000	0.000	0.000	0.124	0.000	0.000
19	Cloppenburg	0.091	0.000	0.000	0.000	0.000	0.000	0.000	0.000
20	Cloppenburg	0.084	0.013	0.000	0.000	0.054	0.327	0.000	0.000
21	Cloppenburg	2.433	0.091	0.000	0.000	0.000	0.000	0.000	0.000
22	Cuxhaven	0.000	0.013	0.000	0.000	0.000	0.024	0.000	0.000
23	Cuxhaven	0.000	0.048	0.000	0.000	0.000	0.000	0.000	0.000
24	Dahme-Spreewald	0.000	0.000	0.000	0.000	0.000	0.000	0.000	0.000
25	Dahme-Spreewald	0.000	0.000	0.000	0.000	0.000	0.000	0.000	0.000
26	Dahme-Spreewald	0.000	0.000	0.000	0.000	0.000	0.000	0.000	0.000
27	Dahme-Spreewald	0.000	0.000	0.000	0.000	0.000	0.000	0.000	0.000
28	Dahme-Spreewald	0.000	0.000	0.000	0.000	0.000	0.000	0.000	0.000
29	Dahme-Spreewald	0.000	0.000	0.000	0.000	0.000	0.000	0.000	0.000
30	Dahme-Spreewald	0.000	0.000	0.000	0.000	0.000	0.000	0.000	0.000
31	Dahme-Spreewald	0.000	0.000	0.000	0.000	0.000	0.000	0.000	0.000

32	Dahme-Spreewald	0.000	0.000	0.000	0.000	0.000	0.000	0.000	0.000
33	Dahme-Spreewald	0.000	0.000	0.000	0.000	0.000	0.000	0.000	0.000
34	Dahme-Spreewald	0.000	0.000	0.000	0.000	0.000	0.000	0.000	0.000
35	Dahme-Spreewald	0.000	0.000	0.000	0.000	0.000	0.000	0.000	0.000
36	Dahme-Spreewald	0.000	0.000	0.000	0.000	0.000	0.000	0.000	0.000
37	Dahme-Spreewald	0.000	0.000	0.000	0.000	0.000	0.000	0.000	0.000
38	Dahme-Spreewald	0.000	0.000	0.000	0.000	0.000	0.000	0.000	0.000
39	Dahme-Spreewald	0.000	0.000	0.000	0.000	0.000	0.000	0.000	0.000
40	Dahme-Spreewald	0.000	0.000	0.000	0.000	0.000	0.000	0.000	0.000
41	Dahme-Spreewald	0.000	0.000	0.000	0.000	0.000	0.000	0.000	0.000
42	Dahme-Spreewald	0.000	0.000	0.000	0.000	0.000	0.000	0.000	0.000
43	Dahme-Spreewald	0.000	0.000	0.000	0.000	0.062	0.000	0.000	0.000
44	Dahme-Spreewald	0.000	0.000	0.000	0.000	0.000	0.000	0.000	0.000
45	Dahme-Spreewald	0.000	0.000	0.000	0.000	0.000	0.000	0.000	0.000
46	Dahme-Spreewald	0.000	0.000	0.000	0.000	0.000	0.000	0.000	0.000
47	Dahme-Spreewald	0.010	0.000	0.000	0.000	0.000	0.000	0.000	0.000
48	Dahme-Spreewald	0.031	0.000	0.000	0.000	0.000	0.068	0.000	0.000
49	Dahme-Spreewald	0.047	0.000	0.000	0.000	0.000	0.000	0.008	0.000
50	Dahme-Spreewald	0.172	0.000	0.000	0.000	0.000	0.000	0.000	0.000
51	Dahme-Spreewald	0.000	0.009	0.000	0.000	0.000	0.000	0.000	0.000
52	Dahme-Spreewald	0.052	0.010	0.000	0.000	0.000	0.000	0.000	0.000
53	Dahme-Spreewald	0.000	0.013	0.000	0.000	0.000	0.000	0.000	0.000
54	Dahme-Spreewald	0.048	0.013	0.000	0.000	0.000	0.000	0.000	0.000
55	Dahme-Spreewald	0.000	0.014	0.013	0.000	0.000	0.000	0.000	0.000
56	Dahme-Spreewald	0.138	0.016	0.000	0.000	0.000	0.000	0.026	0.000
57	Dahme-Spreewald	0.000	0.019	0.000	0.000	0.000	0.000	0.000	0.000
58	Dahme-Spreewald	0.234	0.048	0.000	0.000	0.000	0.000	0.011	0.000
59	Dahme-Spreewald	0.395	0.061	0.000	0.000	0.024	0.022	0.029	0.000
60	Dahme-Spreewald	0.000	0.064	0.000	0.000	0.000	0.000	0.000	0.000
61	Dahme-Spreewald	0.241	0.103	0.000	0.000	0.000	0.000	0.012	0.000
62	Dahme-Spreewald	0.029	0.129	0.000	0.000	0.000	0.000	0.000	0.000
63	Diepholz	0.326	0.061	0.000	0.000	0.000	0.000	0.383	0.000
64	Enzkreis	0.060	0.000	0.000	0.000	0.000	0.000	0.000	0.000
65	Goslar	0.000	0.000	0.000	0.000	0.000	0.000	0.000	0.000

66	Goslar	0.000	0.000	0.000	0.000	0.000	0.000	0.000	0.000
67	Goslar	0.000	0.000	0.000	0.000	0.000	0.000	0.000	0.000
68	Goslar	0.000	0.000	0.000	0.000	0.000	0.000	0.000	0.000
69	Goslar	0.000	0.000	0.000	0.000	0.000	0.000	0.000	0.000
70	Goslar	0.000	0.000	0.000	0.000	0.000	0.000	0.000	0.000
71	Goslar	0.000	0.000	0.000	0.000	0.000	0.000	0.000	0.000
72	Goslar	0.000	0.000	0.000	0.000	0.000	0.000	0.000	0.000
73	Goslar	0.000	0.000	0.000	0.000	0.000	0.000	0.000	0.000
74	Goslar	0.000	0.000	0.000	0.000	0.000	0.000	0.000	0.000
75	Goslar	0.000	0.000	0.000	0.000	0.032	0.000	0.000	0.000
76	Goslar	0.000	0.000	0.000	0.000	0.000	0.000	0.000	0.000
77	Goslar	0.000	0.000	0.000	0.000	0.000	0.000	0.000	0.000
78	Goslar	0.017	0.000	0.000	0.000	0.000	0.088	0.000	0.000
79	Goslar	0.314	0.000	0.000	0.000	0.000	0.000	0.000	0.000
80	Goslar	0.000	0.040	0.000	0.000	0.000	0.000	0.000	0.000
81	Goslar	0.022	0.051	0.000	0.000	0.000	0.000	0.171	0.000
82	Goslar	0.000	0.082	0.000	0.000	0.000	0.000	0.000	0.000
83	Goslar	0.000	0.091	0.000	0.000	0.000	0.000	0.000	0.000
84	Goslar	0.010	0.122	0.000	0.000	0.000	0.000	0.000	0.000
85	Goslar	0.000	0.144	0.000	0.000	0.000	0.000	0.000	0.000
86	Goslar	0.272	0.147	0.000	0.000	0.000	0.000	0.014	0.000
87	Goslar	0.117	0.197	0.000	0.000	0.000	0.000	0.083	0.000
88	Goslar	0.148	1.457	0.000	0.000	0.000	0.000	0.000	0.000
89	Goslar	0.012	1.486	0.000	0.005	0.000	0.000	0.000	0.000
90	Goslar	0.555	1.574	0.000	0.000	0.000	0.000	0.000	0.000
91	Heidelberg	0.178	0.000	0.000	0.000	0.359	0.000	0.000	0.000
92	Helmstedt	0.017	0.860	0.000	0.000	0.012	0.000	0.000	0.000
93	Herford	0.105	0.061	0.000	0.231	0.019	0.171	0.000	0.000
94	Hildesheim	0.000	0.000	0.000	0.000	0.000	0.000	0.000	0.000
95	Höxter	0.000	0.000	0.000	0.000	0.000	0.000	0.000	0.000
96	Höxter	0.014	0.000	0.000	0.000	0.000	0.000	0.000	0.000
97	Höxter	0.014	0.000	0.000	0.020	0.000	0.254	0.000	0.000
98	Höxter	0.291	0.000	0.000	0.000	0.000	0.051	0.077	0.000
99	Höxter	0.697	0.000	0.000	0.000	0.022	0.000	0.000	0.000

100	Höxter	0.060	0.057	0.000	0.000	0.059	0.000	0.000	0.000
101	Karlsruhe	0.053	0.012	0.000	0.000	0.000	0.000	0.018	0.000
102	Karlsruhe	0.053	0.012	0.000	0.000	0.000	0.000	0.018	0.000
103	Leer	0.000	0.000	0.000	0.000	0.029	0.000	0.000	0.000
104	Leer	0.436	0.040	0.000	0.000	0.029	0.000	0.000	0.000
105	Leer	0.112	0.612	0.000	0.000	0.000	0.000	0.000	0.000
106	Lippe	0.000	0.000	0.000	0.000	0.000	0.000	0.000	0.000
107	Lippe	0.000	0.000	0.000	0.000	0.000	0.000	0.000	0.000
108	Lippe	0.000	0.000	0.000	0.000	0.000	0.000	0.000	0.000
109	Lippe	0.041	0.000	0.000	0.000	0.000	0.024	0.000	0.000
110	Lippe	0.043	0.000	0.000	0.000	0.022	0.000	0.109	0.000
111	Lippe	0.045	0.000	0.000	0.000	0.022	0.000	0.000	0.000
112	Lippe	0.060	0.000	0.000	0.114	0.000	0.037	0.000	0.000
113	Lippe	0.147	0.000	0.000	0.000	0.000	0.000	0.000	0.000
114	Lippe	0.447	0.000	0.000	0.891	0.013	0.000	0.000	0.000
115	Lippe	1.184	0.000	0.000	0.350	0.000	0.000	0.000	0.000
116	Lippe	0.222	0.014	0.000	0.000	0.028	0.037	0.000	0.000
117	Lippe	0.469	0.022	0.000	0.000	0.082	0.000	0.000	0.000
118	Lippe	0.821	0.047	0.000	0.000	0.000	0.000	0.000	0.000
119	Lippe	0.697	0.048	0.000	0.090	0.063	0.178	0.123	0.000
120	Lippe	0.043	0.069	0.000	0.000	0.026	0.000	0.000	0.000
121	Lippe	0.021	0.088	0.000	0.000	0.000	0.049	0.000	0.000
122	Lippe	0.188	0.122	0.000	0.000	0.000	0.190	0.000	0.000
123	Lippe	0.034	0.155	0.000	0.030	0.196	0.000	0.017	0.000
124	Lippe	1.172	0.184	0.000	0.000	0.021	0.051	0.055	0.000
125	Lippe	0.000	0.190	0.000	0.019	0.000	0.000	0.000	0.000
126	Lippe	0.000	1.375	0.000	0.000	0.000	0.000	0.000	0.000
127	Lüneburg	0.166	0.000	0.000	0.000	0.000	0.000	0.000	0.000
128	Minden-Lübbecke	0.090	0.000	0.000	0.000	0.774	0.000	0.000	0.000
129	Minden-Lübbecke	0.934	0.021	0.000	0.000	0.094	0.022	0.838	0.000
130	Minden-Lübbecke	0.412	0.053	0.000	0.000	0.019	0.146	0.062	0.000
131	Minden-Lübbecke	0.131	0.070	0.000	0.000	0.000	0.000	0.000	0.000
132	Minden-Lübbecke	0.617	0.257	0.000	0.000	0.035	0.000	0.000	0.000
133	Münster	0.000	0.000	0.000	0.000	0.000	0.000	0.000	0.000

134	Neckar-Odenwald-Kreis	0.000	0.000	0.000	0.000	0.000	0.000	0.000	0.000
135	Neckar-Odenwald-Kreis	0.000	0.000	0.000	0.000	0.000	0.000	0.000	0.000
136	Neckar-Odenwald-Kreis	0.000	0.000	0.000	0.000	0.000	0.000	0.000	0.000
137	Neckar-Odenwald-Kreis	0.000	0.000	0.000	0.000	0.000	0.000	0.000	0.000
138	Neckar-Odenwald-Kreis	0.000	0.000	0.000	0.000	0.000	0.000	0.000	0.000
139	Neckar-Odenwald-Kreis	0.000	0.000	0.000	0.000	0.000	0.000	0.000	0.000
140	Neckar-Odenwald-Kreis	0.000	0.000	0.000	0.000	0.000	0.000	0.000	0.000
141	Neckar-Odenwald-Kreis	0.000	0.000	0.000	0.000	0.000	0.000	0.000	0.000
142	Neckar-Odenwald-Kreis	0.000	0.000	0.000	0.000	0.000	0.000	0.000	0.000
143	Neckar-Odenwald-Kreis	0.000	0.000	0.000	0.000	0.000	0.000	0.000	0.000
144	Neckar-Odenwald-Kreis	0.000	0.000	0.000	0.000	0.000	0.000	0.000	0.000
145	Neckar-Odenwald-Kreis	0.000	0.000	0.000	0.000	0.000	0.000	0.000	0.000
146	Neckar-Odenwald-Kreis	0.000	0.000	0.000	0.000	0.000	0.000	0.000	0.000
147	Neckar-Odenwald-Kreis	0.000	0.000	0.000	0.000	0.000	0.000	0.000	0.000
148	Neckar-Odenwald-Kreis	0.000	0.000	0.000	0.000	0.000	0.000	0.000	0.000
149	Neckar-Odenwald-Kreis	0.000	0.000	0.000	0.000	0.000	0.000	0.000	0.000
150	Neckar-Odenwald-Kreis	0.000	0.000	0.000	0.000	0.000	0.000	0.000	0.000
151	Neckar-Odenwald-Kreis	0.000	0.000	0.000	0.000	0.000	0.000	0.000	0.000
152	Neckar-Odenwald-Kreis	0.000	0.000	0.000	0.000	0.000	0.000	0.000	0.000
153	Neckar-Odenwald-Kreis	0.019	0.000	0.000	0.000	0.000	0.000	0.000	0.000
154	Neckar-Odenwald-Kreis	0.043	0.000	0.000	0.000	0.000	0.000	0.000	0.000
155	Neckar-Odenwald-Kreis	0.045	0.000	0.000	0.000	0.000	0.000	0.000	0.000
156	Neckar-Odenwald-Kreis	0.048	0.000	0.000	0.000	0.000	0.000	0.000	0.000
157	Neckar-Odenwald-Kreis	0.088	0.000	0.000	0.000	0.010	0.000	0.000	0.000
158	Neckar-Odenwald-Kreis	0.098	0.000	0.000	0.000	0.000	0.000	0.000	0.000
159	Neckar-Odenwald-Kreis	0.164	0.000	0.000	0.000	0.000	0.000	0.000	0.000
160	Neckar-Odenwald-Kreis	0.028	0.044	0.000	0.000	0.000	0.000	0.000	0.000
161	Neckar-Odenwald-Kreis	0.574	0.045	0.000	0.000	0.000	0.000	0.000	0.000
162	Neckar-Odenwald-Kreis	0.038	0.256	0.000	0.000	0.000	0.000	0.072	0.000
163	Nienburg (Weser)	0.222	0.871	0.000	0.000	0.000	0.000	0.038	0.000
164	Oberhavel	0.000	0.000	0.000	0.000	0.000	0.000	0.042	0.000
165	Oberhavel	0.000	0.000	0.000	0.000	0.000	0.000	0.000	0.000
166	Oberhavel	0.000	0.000	0.000	0.000	0.000	0.000	0.000	0.000
167	Oberhavel	0.000	0.000	0.000	0.000	0.000	0.000	0.000	0.000

168	Oberhavel	0.000	0.000	0.000	0.000	0.000	0.000	0.000	0.000
169	Oberhavel	0.000	0.000	0.000	0.000	0.000	0.000	0.000	0.000
170	Oberhavel	0.000	0.000	0.000	0.000	0.000	0.000	0.000	0.000
171	Oberhavel	0.000	0.000	0.000	0.000	0.000	0.000	0.008	0.000
172	Oberhavel	0.000	0.000	0.000	0.000	0.000	0.000	0.000	0.000
173	Oberhavel	0.000	0.000	0.000	0.000	0.000	0.000	0.000	0.000
174	Oberhavel	0.000	0.000	0.000	0.000	0.000	0.000	0.000	0.000
175	Oberhavel	0.000	0.000	0.000	0.000	0.000	0.000	0.000	0.000
176	Oberhavel	0.000	0.000	0.000	0.000	0.000	0.000	0.000	0.000
177	Oberhavel	0.000	0.000	0.000	0.000	0.000	0.000	0.320	0.000
178	Oberhavel	0.000	0.000	0.000	0.000	0.000	0.000	0.000	0.000
179	Oberhavel	0.000	0.000	0.000	0.000	0.000	0.000	0.000	0.000
180	Oberhavel	0.000	0.000	0.000	0.000	0.000	0.000	0.000	0.000
181	Oberhavel	0.000	0.000	0.000	0.000	0.000	0.000	0.000	0.000
182	Oberhavel	0.000	0.000	0.000	0.000	0.000	0.000	0.000	0.000
183	Oberhavel	0.000	0.000	0.000	0.000	0.000	0.000	0.000	0.000
184	Oberhavel	0.000	0.000	0.000	0.000	0.000	0.000	0.000	0.000
185	Oberhavel	0.000	0.000	0.000	0.000	0.000	0.000	0.000	0.000
186	Oberhavel	0.012	0.000	0.000	0.000	0.000	0.000	0.000	0.000
187	Oberhavel	0.014	0.000	0.000	0.000	0.000	0.000	0.000	0.000
188	Oberhavel	0.017	0.000	0.000	0.000	0.015	0.000	0.000	0.000
189	Oberhavel	0.028	0.000	0.000	0.000	0.000	0.000	0.000	0.000
190	Oberhavel	0.086	0.000	0.000	0.000	0.000	0.000	0.000	0.000
191	Oberhavel	0.157	0.000	0.000	0.000	0.050	0.000	0.000	0.000
192	Oberhavel	0.202	0.000	0.000	0.000	0.000	0.000	0.000	0.000
193	Oberhavel	0.336	0.000	0.000	0.000	0.000	0.000	0.000	0.000
194	Oberhavel	0.690	0.000	0.000	0.000	0.000	0.000	0.008	0.000
195	Oberhavel	0.000	0.006	0.000	0.000	0.000	0.000	0.008	0.000
196	Oberhavel	0.053	0.006	0.000	0.000	0.000	0.000	0.000	0.000
197	Oberhavel	0.060	0.030	0.000	0.000	0.171	0.000	0.000	0.000
198	Oberhavel	0.000	0.034	0.000	0.000	0.423	0.000	0.000	0.000
199	Oberhavel	0.000	0.040	0.000	0.000	0.000	0.000	0.000	0.000
200	Oberhavel	0.109	0.042	0.000	0.000	0.000	0.000	0.000	0.000
201	Oberhavel	0.034	0.081	0.000	0.000	0.000	0.000	0.038	0.000

202	Oberhavel	0.347	0.155	0.000	0.000	0.000	0.000	0.000	0.000
203	Oberhavel	0.028	0.357	0.000	0.000	0.044	0.000	0.000	0.000
204	Oberhavel	0.847	0.810	0.000	0.003	0.000	0.000	0.103	0.000
205	Oldenburg	0.000	0.000	0.000	0.000	0.000	0.000	0.000	0.000
206	Oldenburg	0.012	0.000	0.000	0.000	0.000	0.000	0.000	0.000
207	Oldenburg	0.188	0.018	0.000	0.000	0.000	0.000	0.000	0.000
208	Oldenburg	0.274	0.062	0.000	0.000	0.000	0.000	0.000	0.000
209	Oldenburg	0.017	0.084	0.000	0.000	0.000	0.000	0.085	0.000
210	Oldenburg	0.279	0.123	0.000	0.141	0.000	0.000	0.000	0.000
211	Oldenburg	0.012	0.156	0.000	0.000	0.000	0.000	0.074	0.000
212	Oldenburg	0.147	0.386	0.000	0.000	0.000	0.000	0.000	0.000
213	Paderborn	0.000	0.000	0.000	0.025	0.000	0.000	0.000	0.000
214	Paderborn	0.000	0.000	0.000	0.000	0.000	0.000	0.000	0.000
215	Rastatt	0.000	0.000	0.000	0.000	0.000	0.000	0.000	0.000
216	Region Hannover	0.000	0.000	0.000	0.000	0.000	0.000	0.000	0.000
217	Rems-Murr-Kreis	0.000	0.000	0.000	0.000	0.000	0.000	0.000	0.000
218	Rhein-Neckar-Kreis	0.000	0.000	0.000	0.000	0.000	0.000	0.105	0.000
219	Rhein-Neckar-Kreis	0.000	0.000	0.000	0.000	0.000	0.000	0.000	0.000
220	Rhein-Neckar-Kreis	0.000	0.000	0.000	0.000	0.000	0.000	0.000	0.000
221	Rhein-Neckar-Kreis	0.000	0.000	0.000	0.000	0.000	0.000	0.000	0.000
222	Rhein-Neckar-Kreis	0.000	0.000	0.000	0.000	0.000	0.000	0.000	0.000
223	Rhein-Neckar-Kreis	0.000	0.000	0.000	0.000	0.000	0.000	0.000	0.000
224	Rhein-Neckar-Kreis	0.000	0.000	0.000	0.000	0.000	0.000	0.000	0.000
225	Rhein-Neckar-Kreis	0.000	0.000	0.000	0.000	0.072	0.000	0.000	0.000
226	Rhein-Neckar-Kreis	0.000	0.000	0.000	0.000	0.000	0.000	0.000	0.000
227	Rhein-Neckar-Kreis	0.000	0.000	0.000	0.000	0.000	0.000	0.000	0.000
228	Rhein-Neckar-Kreis	0.016	0.000	0.000	0.000	0.000	0.000	0.026	0.000
229	Rhein-Neckar-Kreis	0.017	0.000	0.000	0.099	0.000	0.054	0.038	0.000
230	Rhein-Neckar-Kreis	0.017	0.000	0.000	0.000	0.000	0.000	0.000	0.000
231	Rhein-Neckar-Kreis	0.019	0.000	0.000	0.000	0.000	0.000	0.000	0.000
232	Rhein-Neckar-Kreis	0.026	0.000	0.000	0.000	0.000	0.000	0.000	0.000
233	Rhein-Neckar-Kreis	0.031	0.000	0.000	0.000	0.000	0.000	0.000	0.000
234	Rhein-Neckar-Kreis	0.131	0.000	0.000	0.000	0.000	0.000	0.000	0.000
235	Rhein-Neckar-Kreis	0.028	0.052	0.000	0.000	0.000	0.000	0.000	0.000

236	Rhein-Neckar-Kreis	0.000	0.065	0.000	0.000	0.000	0.000	0.100	0.000
237	Rhein-Neckar-Kreis	1.943	0.086	0.000	0.000	0.000	0.000	0.000	0.000
238	Rhein-Neckar-Kreis	0.000	0.113	0.000	0.000	0.000	0.000	0.000	0.000
239	Rhein-Neckar-Kreis	0.884	0.145	0.000	0.000	0.000	0.207	0.000	0.000
240	Rhein-Neckar-Kreis	0.000	0.192	0.000	0.000	0.000	0.117	0.092	0.000
241	Rhein-Neckar-Kreis	0.053	0.230	0.000	0.000	0.000	0.000	0.075	0.000
242	Rhein-Neckar-Kreis	0.000	0.240	0.000	0.000	0.000	0.049	0.000	0.000
243	Rhein-Neckar-Kreis	0.028	0.400	0.000	0.008	0.017	0.112	0.000	0.000
244	Rhein-Neckar-Kreis	0.053	1.116	0.000	0.012	0.021	0.000	0.000	0.000
245	Rhein-Neckar-Kreis	0.097	1.179	0.000	0.000	0.000	0.017	0.018	0.000
246	Uckermark	0.000	0.000	0.000	0.000	0.000	0.000	0.000	0.000
247	Uckermark	0.000	0.000	0.000	0.000	0.000	0.000	0.000	0.000
248	Uckermark	0.000	0.000	0.000	0.000	0.000	0.000	0.000	0.000
249	Uckermark	0.000	0.000	0.000	0.000	0.000	0.000	0.000	0.000
250	Uckermark	0.000	0.000	0.000	0.000	0.000	0.000	0.000	0.000
251	Uckermark	0.000	0.000	0.000	0.000	0.000	0.000	0.000	0.000
252	Uckermark	0.000	0.000	0.000	0.000	0.000	0.000	0.000	0.000
253	Uckermark	0.000	0.000	0.000	0.000	0.000	0.000	0.000	0.000
254	Uckermark	0.000	0.000	0.000	0.000	0.000	0.000	0.000	0.000
255	Uckermark	0.000	0.000	0.000	0.000	0.000	0.000	0.000	0.000
256	Uckermark	0.000	0.000	0.000	0.000	0.000	0.000	0.000	0.000
257	Uckermark	0.000	0.000	0.000	0.000	0.000	0.000	0.000	0.000
258	Uckermark	0.000	0.000	0.000	0.000	0.000	0.000	0.000	0.000
259	Uckermark	0.000	0.000	0.000	0.000	0.000	0.000	0.000	0.000
260	Uckermark	0.000	0.000	0.000	0.000	0.000	0.000	0.000	0.000
261	Uckermark	0.000	0.000	0.000	0.000	0.000	0.000	0.000	0.000
262	Uckermark	0.012	0.000	0.000	0.000	0.000	0.000	0.000	0.000
263	Uckermark	0.019	0.000	0.000	0.000	0.015	0.000	0.000	0.000
264	Uckermark	0.081	0.000	0.000	0.000	0.000	0.000	0.115	0.000
265	Uckermark	0.162	0.000	0.000	0.000	0.000	0.000	0.000	0.000
266	Uckermark	0.012	0.004	0.000	0.000	0.000	0.000	0.000	0.000
267	Uckermark	0.000	0.013	0.000	0.000	0.000	0.000	0.000	0.000
268	Uckermark	0.164	0.014	0.000	0.000	0.000	0.000	0.000	0.012
269	Uckermark	0.019	0.022	0.000	0.000	0.000	0.000	0.000	0.000

270	Uckermark	0.000	0.023	0.000	0.000	0.000	0.000	0.283	0.000
271	Uckermark	0.000	0.026	0.000	0.000	0.000	0.000	0.000	0.000
272	Uckermark	0.736	0.030	0.000	0.000	0.000	0.000	0.000	0.000
273	Uckermark	0.000	0.035	0.000	0.000	0.000	0.000	0.000	0.000
274	Uckermark	0.000	0.044	0.000	0.000	0.000	0.000	0.000	0.000
275	Uckermark	0.088	0.055	0.000	0.000	0.000	0.061	0.000	0.000
276	Uckermark	0.169	0.081	0.000	0.000	0.000	0.090	0.000	0.000
277	Uckermark	0.000	0.088	0.000	0.000	0.000	0.000	0.400	0.000
278	Uckermark	0.050	0.096	0.000	0.000	0.000	0.000	0.000	0.000
279	Uckermark	0.000	0.132	0.000	0.000	0.000	0.000	0.000	0.000
280	Uckermark	0.033	0.200	0.000	0.000	0.014	0.000	0.000	0.000
281	Uckermark	0.429	0.275	0.000	0.000	0.000	0.000	0.028	0.000
282	Uckermark	1.890	0.416	0.000	0.000	0.000	0.000	0.000	0.000
283	Uckermark	0.314	0.490	0.000	0.000	0.000	0.000	0.140	0.000
284	Uckermark	0.859	0.525	0.000	0.000	0.000	0.000	0.038	0.000
285	Vechta	0.481	0.016	0.000	0.000	0.000	0.000	0.000	0.008
286	Vechta	0.307	0.027	0.000	0.000	0.000	0.000	0.000	0.000
287	Verden	0.000	0.000	0.000	0.000	0.000	0.000	0.000	0.000
288	Warendorf	0.000	0.000	0.000	0.000	0.000	0.000	0.000	0.000
289	Warendorf	0.000	0.000	0.000	0.000	0.000	0.000	0.000	0.000
290	Warendorf	0.000	0.000	0.000	0.000	0.000	0.000	0.000	0.000
291	Warendorf	0.000	0.000	0.000	0.000	0.000	0.000	0.000	0.000
292	Warendorf	0.000	0.000	0.000	0.000	0.000	0.000	0.000	0.000
293	Warendorf	0.000	0.000	0.000	0.000	0.000	0.000	0.000	0.000
294	Warendorf	0.000	0.000	0.000	0.002	0.000	0.000	0.025	0.000
295	Warendorf	0.021	0.000	0.000	0.000	0.000	0.000	0.000	0.000
296	Warendorf	0.050	0.000	0.000	0.000	0.000	0.000	0.000	0.000
297	Warendorf	0.055	0.000	0.000	0.000	0.000	0.000	0.000	0.000
298	Warendorf	0.488	0.000	0.000	0.000	0.000	0.000	0.000	0.000
299	Warendorf	0.517	0.000	0.000	0.000	0.000	0.000	0.000	0.000
300	Warendorf	0.000	0.010	0.000	0.000	0.000	0.000	0.000	0.000
301	Warendorf	0.000	0.010	0.000	0.000	0.000	0.000	0.038	0.000
302	Warendorf	0.040	0.012	0.000	0.000	0.000	0.000	0.000	0.000
303	Warendorf	0.000	0.014	0.000	0.000	0.000	0.000	0.000	0.000

304	Warendorf	0.000	0.018	0.000	0.001	0.000	0.000	0.000	0.000
305	Warendorf	2.203	0.025	0.000	0.000	0.000	0.000	0.000	0.000
306	Warendorf	0.267	0.032	0.000	0.000	0.000	0.000	0.000	0.000
307	Warendorf	0.267	0.032	0.000	0.000	0.000	0.000	0.000	0.000
308	Warendorf	0.000	0.035	0.000	0.000	0.000	0.000	0.000	0.000
309	Warendorf	0.000	0.036	0.000	0.000	0.000	0.000	0.000	0.000
310	Warendorf	0.924	0.047	0.000	0.000	0.000	0.000	0.000	0.000
311	Warendorf	0.507	0.053	0.000	0.000	0.000	0.000	0.035	0.000
312	Warendorf	1.379	0.079	0.000	0.014	0.000	0.000	0.000	0.000
313	Warendorf	0.634	0.082	0.000	0.000	0.000	0.000	0.000	0.000
314	Warendorf	0.652	0.130	0.000	0.000	0.000	0.000	0.000	0.000
315	Warendorf	0.605	0.171	0.000	0.000	0.000	0.000	0.000	0.000
316	Warendorf	0.847	0.323	0.000	0.000	0.000	0.000	0.000	0.000
317	Wolfenbüttel	0.000	0.000	0.000	0.000	0.000	0.000	0.000	0.000
318	Wolfenbüttel	0.000	0.000	0.000	0.000	0.000	0.000	0.000	0.000
319	Wolfenbüttel	0.000	0.000	0.000	0.000	0.000	0.000	0.000	0.000
320	Wolfenbüttel	0.000	0.000	0.000	0.000	0.000	0.000	0.000	0.000
321	Wolfenbüttel	0.000	0.000	0.000	0.408	0.000	0.000	0.298	0.000
322	Wolfenbüttel	0.000	0.000	0.000	0.000	0.000	0.000	0.000	0.000
323	Wolfenbüttel	0.000	0.000	0.000	0.000	0.000	0.000	0.000	0.000
324	Wolfenbüttel	0.000	0.000	0.000	0.000	0.000	0.000	0.000	0.000
325	Wolfenbüttel	0.000	0.000	0.000	0.000	0.000	0.000	0.000	0.000
326	Wolfenbüttel	0.000	0.000	0.000	0.000	0.000	0.000	0.000	0.000
327	Wolfenbüttel	0.000	0.000	0.000	0.000	0.228	0.000	0.000	0.000
328	Wolfenbüttel	0.097	0.000	0.000	0.000	0.000	0.000	0.000	0.000
329	Wolfenbüttel	0.014	0.013	0.000	0.000	0.000	0.000	0.000	0.000
330	Wolfenbüttel	0.000	0.014	0.000	0.000	0.000	0.000	0.000	0.000
331	Wolfenbüttel	0.000	0.040	0.000	0.000	0.000	0.000	0.000	0.000
